# Mediating Role of Activity Level in the Depressive Realism Effect

**DOI:** 10.1371/journal.pone.0046203

**Published:** 2012-09-27

**Authors:** Fernando Blanco, Helena Matute, Miguel A. Vadillo

**Affiliations:** Departamento de Fundamentos y Métodos de la Psicología, University of Deusto, Bilbao, Spain; Peking University, China

## Abstract

Several classic studies have concluded that the accuracy of identifying uncontrollable situations depends heavily on depressive mood. Nondepressed participants tend to exhibit an optimistic illusion of control, whereas depressed participants tend to better detect a lack of control. Recently, we suggested that the different activity levels (measured as the probability of responding during a contingency learning task) exhibited by depressed and nondepressed individuals is partly responsible for this effect. The two studies presented in this paper provide further support for this mediational hypothesis, in which mood is the distal cause of the illusion of control operating through activity level, the proximal cause. In Study 1, the probability of responding, P(R), was found to be a mediator variable between the depressive symptoms and the judgments of control. In Study 2, we intervened directly on the mediator variable: The P(R) for both depressed and nondepressed participants was manipulated through instructions. Our results confirm that P(R) manipulation produced differences in the participants’ perceptions of uncontrollability. Importantly, the intervention on the mediator variable cancelled the effect of the distal cause; the participants’ judgments of control were no longer mood dependent when the P(R) was manipulated. This result supports the hypothesis that the so-called depressive realism effect is actually mediated by the probability of responding.

## Introduction

Far from being optimal reasoners, people are known to exhibit certain cognitive biases or systematic errors when performing certain tasks. One of the most extensively studied cognitive biases is the illusion of control, which consists of a belief that one is able to exert control over outcomes that are actually uncontrollable. This effect occurs mainly when the outcomes are of appetitive valence and occur frequently [1]–[3].

A very influential paper by Alloy and Abramson [1] showed that the illusion of control was stronger in nondepressed participants. In that study, both nondepressed and mildly depressed students were allowed to decide during each trial whether to press a button to turn on a light bulb. The light bulb’s onset was actually independent of the pressing of the button (i.e., it was uncontrollable). During a training phase, the participants were able to press the button as often as they wanted, and the light came on during some of the trials but not others. Afterward, the participants were asked about the degree of control they had exerted over the light onset; that is, they were asked about the perceived contingency between their responses and the outcomes. Interestingly, Alloy and Abramson [1] found that mildly depressed participants were better at detecting the absence of control over the light than nondepressed participants were; nondepressed participants systematically overestimated the contingency between their actions and the uncontrollable outcome, thereby exhibiting an illusion of control [4]–[6]. Alloy and Abramson’s finding soon became known as “depressive realism” [7] and has been replicated several times using different tasks and procedures [8]–[12].

A variety of hypotheses have been proposed to account for this puzzling finding (see a review by Ackerman & DeRubeis [13], and recent proposals by different authors [9]–[11], [14]). An examination of the published literature leads to the impression that the presumed realism of depressed people may be a complex, multicausal phenomenon that involves many variables and demands further research.

One variable that has been shown to be a source of bias in contingency judgments is the activity level of the participants, as determined by the response frequency [6], [15], [16]. Participants who take an active approach to this type of experiment become exposed to frequent, yet accidental, pairings between their responses and the uncontrollable outcome. These co-occurrences lead them to believe that they are indeed causing the outcome; as a result, they develop an illusion of control.

Inspired by this rationale, a recent study by Blanco et al. [9] suggested that the passivity associated with depressive symptoms does, to some extent, contribute to realism in detecting the uncontrollability in the standard contingency-learning tasks normally used in studies of depressive realism [1]. According to Blanco et al. [9], nondepressed participants tend to spontaneously respond more frequently during the training phase. This means that in a typical contingency learning task, nondepressed participants decide to press the button on more trials than do depressed participants, who very often take a more passive approach to the task [17]. This passive strategy leads to few accidental co-occurrences between the response and the outcome and thus might prevent the development of an illusion of control. In line with these predictions, Blanco et al. showed that mildly depressed participants were both more passive and more realistic than nondepressed participants in an experimental task involving uncontrollable outcomes, similar to that of Alloy and Abramson [1]. In addition, the authors also reported that passivity was correlated with accuracy in detecting the absence of control. The latter result converges with Matute’s [6] findings that the more active participants develop stronger illusions of control, although Matute did not include a measure of depressive symptoms in her study. As a conclusion, Blanco et al. [9] suggested that the depressive realism effect could partially be due to the passivity that depressive participants exhibit in a variety of experimental settings. Thus, depressive symptoms would be the distal cause of the realistic detection of uncontrollability via a more proximal cause, depressive passivity, defined as a lower probability of responding during a contingency learning task.

Nonetheless, the claim that passivity associated with depressive symptoms plays a relevant causal role in the depressive realism effect needs to be supported by careful data analysis and, more importantly, by evidence from experimental designs in which the appropriate manipulations are carried out. Thus, our current aim is to complement the work started by Blanco et al. [9] by using two strategies, statistical and experimental. First, we conducted an exact replication of Blanco et al.’s study [9], but we included a proper mediational analysis. This statistical technique is intended to test the hypothesis by isolating the direct effect of depressive symptoms on control judgments while partialling out the effect of the probability of responding, P(R). In the second study, we conducted an experimental manipulation to further test the mediational hypothesis. Specifically, we intervened directly on the mediator variable, P(R). Because the hypothesized proximal cause of the illusion, P(R), was manipulated externally, if the mediational hypothesis were true, we would observe that the hypothesized distal cause (i.e., depressive symptoms) and the outcome variable (i.e., control judgments) become mutually independent. In other words, we expect that the depressive realism effect should disappear in our experimental study when we directly intervene on the mediator between depressive symptoms and control judgments. The use of this intervention strategy to reveal causal links is described in the causal reasoning literature [18], [19] as based on the Markov condition for Bayesian networks, which states that a variable is probabilistically independent of any other variable (except for its descendants) conditional on its parents. In our case, if the mediational model is true, intervening on the parent of the outcome variable (i.e., its proximal cause) will render the outcome variable and the distal cause independent from each other.

## Study 1

Study 1 was an exact replication of Blanco et al. [9]. Therefore, we expect that participants who are more depressed would show more passive behaviors (i.e., fewer responses) during the training phase and would exhibit more accurate and realistic control judgments. This prediction stems from previous findings and from our interpretation of the depressive realism effect as a consequence of the lower probability of responding. Importantly, Study 1 features a more appropriate statistical procedure than the one that Blanco et al. [9] used. Specifically, in the current study we make use of a mediation analysis aimed at testing whether the probability of responding is a significant mediator between depressive symptoms and control judgments.

### Methods

#### Participants and apparatus

Fifty Psychology students at the University of Deusto agreed to participate in the study as an optional and voluntary activity in their course. The students were not asked to give their name or any other personal information, and were given neither course credit nor monetary compensation for this activity. Data from one participant were excluded because he/she did not respond on any trial, thus making it impossible to judge whether he/she was paying attention. The study took place during a collective session in a large computer room, and was carried out by a teaching assistant different from the course provider.

The study used the Flashes task, programmed in JavaScript. Previous studies have demonstrated that this task is particularly suitable for assessing the illusion of control [20] and the depressive realism effect [9] both on the Internet and in the laboratory.

#### Ethics statement

In Study 1 and Study 2, the behavioral and verbal data generated by the participants were sent anonymously to the experimenters’ e-mail address through the Internet browser. In agreement with the ethical standards for human research on the Internet [21], [22], the participants were informed before the session that in no case they would be asked to provide any personal information (e.g., name, phone, address, e-mail), that their data would not be identifiable, and that they would be allowed to terminate the study by closing the Internet browser window at any moment without penalty, if they wished so. In addition, right after the study finished, a screen requested the participants to give permission to use the data they had just generated. The participants agreed to grant this permission by clicking a button labeled “Send data”, which immediately submitted the data anonymously to the experimenters’ database. Those participants not willing to send the information after the experiment were instructed to click a button labeled “Finish”, which immediately deleted the data. Finally, we did not use cookies, keyloggers or any other software to covertly obtain information from the participants.

The funding agency for this research (i.e., Spanish Ministry of Science and Innovation) evaluated the scientific and practical implications of the procedure used in these studies prior to the approval of the research grant, and did not request any extra formal evaluation from an ethical review board (neither before the allocation of funds nor in subsequent renewals). This was in agreement with the code of ethics of the Spanish Psychological Association [23] (Chapter IV, Articles 33 to 38), which does not state the compulsory nature of formal ethical approval before carrying out this type of innocuous psychological research. In our procedure, the data that the participants provided were anonymous and unidentifiable, the stimuli and materials were harmless and emotionally neutral, the goal of the study was transparent, and the task involved no deception.

#### Procedure

The procedure was identical to the one used by Blanco et al. [9]. A computerized version of Beck’s Depression Inventory (BDI [24]; Spanish adaptation by Conde & Useros [25]) was administered prior to the study, in order to assess the participants’ depressive symptoms. Rather than establishing an arbitrary cutoff point to split the sample into mildly depressed and nondepressed participants, as has usually been done in previous studies [1], [9]–[11], the direct BDI score was taken as a continuous variable. Acknowledging the actual nature of the variable, which is indeed continuous, permits a less biased and more powerful approach to data analysis (for a more elaborated argument on this methodological issue, see Cohen [26]). Using BDI scores as a continuous dimension, rather than as a categorical one, raises a terminology-related issue that must be addressed. Given that our sample is not clinical, the term “depressed” seems inadequate. However, once we acknowledge that our measure of depressive symptoms is continuous in nature, it seems reasonable to use the term “depressed” not as a clinical label, but as an indicator of a given participant’s or group’s higher BDI scores relative to others in the sample. Therefore, one may say that one participant is “more depressed” than other, while none of them is clinically depressed. Thus, through the manuscript, we use the terms “depressed” and “nondepressed” not as indicators of a clinical diagnosis, but as a indicators of relative individual or group differences in the measure of the depressive symptoms.

After recording the BDI score, the program displayed written instructions for the task. The participants were told that a flash would appear on the screen from time to time and last for 1 second. Then, the screen would fade to black. As the instructions described, the participants’ goal was to make the flash appear again whenever it stopped by pressing the space bar on the keyboard. That is, the termination of the flash signaled the opportunity to respond to make it appear again. After the instructions, a series of fifty flashes with a fixed duration of 1 second was presented, interspersed with fifty black-screen intervals. The lengths of these black intervals were programmed to be either short (1 second) or long (5 seconds) according to a prescheduled randomized sequence. This means that, on a given trial during which the participant had responded (by pressing the space bar) right after the previous flash stopped, the flash could follow either immediately (i.e., with a short, 1-second interval), thus leading to an accidental concurrence between the response and the flash, or after a delay (i.e., a long, 5-second interval). The instructions specified that the flashes would appear after a short interval of 1 second whenever the participant succeeded at producing them. They also specified that the flashes would appear after a delay of 5 seconds whenever the participant failed. In other words, the trials in which the flash appears after a short 1-second interval are given reinforcing value through instructions, whereas the trials in which the flash appears after a long delay (5 seconds) are intended to serve as unreinforced trials.

Given that the sequence of flashes was prescheduled and therefore response-independent, the onset of the flashes was actually uncontrollable for the participants. Nevertheless, accidental concurrences between response and flash were expected. These concurrences, if frequent, could lead to the impression that flashes were under the participants’ control. The fact that 38 out of 50 trials (76%) included a short black interval and only 12 trials (24%) included a long black interval (i.e., 76% of trials were potentially reinforced) was aimed at favoring the illusion of control, based on previous studies showing that a high probability of reinforcement produces stronger illusions [1], [2]. The predefined sequence of trials was identical to that used by Blanco et al. [9] to render a fair comparison of the two studies.

The activity level of every participant was measured by computing the probability of responding, P(R), during the training phase. This value results from dividing the number of trials in which a response (a key press) was detected by the total number of trials (50), hence the variable ranges between 0 and 1.

Finally, the participants’ perceived control over the flashes was assessed via a control judgment after the training phase. The test question, translated from the original in Spanish, read as follows: “To what extent do you think that the onset of the flashes depended on what you did?” The answers were given by clicking on a scale ranging from 0 (“Absolutely not”) to 100 (“Absolutely”). Given that the flashes were presented in a preprogrammed order, one can assume that any judgment higher than zero was biased up to a certain extent.

### Results and Discussion

**Figure 1:**
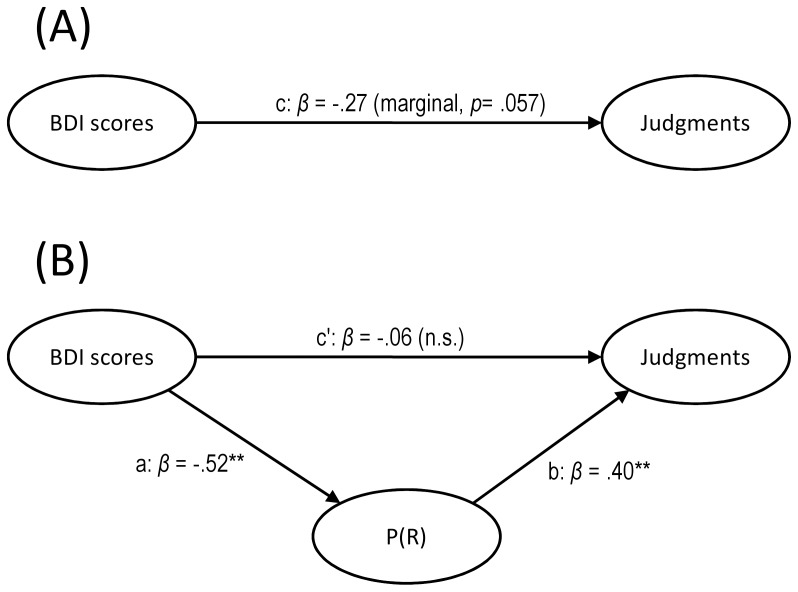
Mediational model tested in Study 1. The letters a, b, c, and c’ depict the paths between the three variables, which are weighted by the standardized regression coefficients. One or two asterisks indicate a significant coefficient (p<.05 or p<.01, respectively), while n.s. means that the coefficient failed to reach the significance level (p>.05). Panel (A): Path c corresponds to the predictive link between BDI scores and the judgments (i.e., the total effect in the model). The negative coefficient indicates that we replicated the depressive realism effect. Panel (B): The total effect (Path c above) can be decomposed into two components, the direct effect and the indirect effect. Paths a and b represent the indirect effect that operates via the mediator, P(R). Path c’ represents the direct effect of BDI scores on the judgments, that is, the amount of predictive power left after the mediational effect of P(R) has been partialed out. Overall, the mediational analyses support a causal chain model in which BDI scores produce depressive realism indirectly via the mediator variable, P(R).

**Figure 2:**
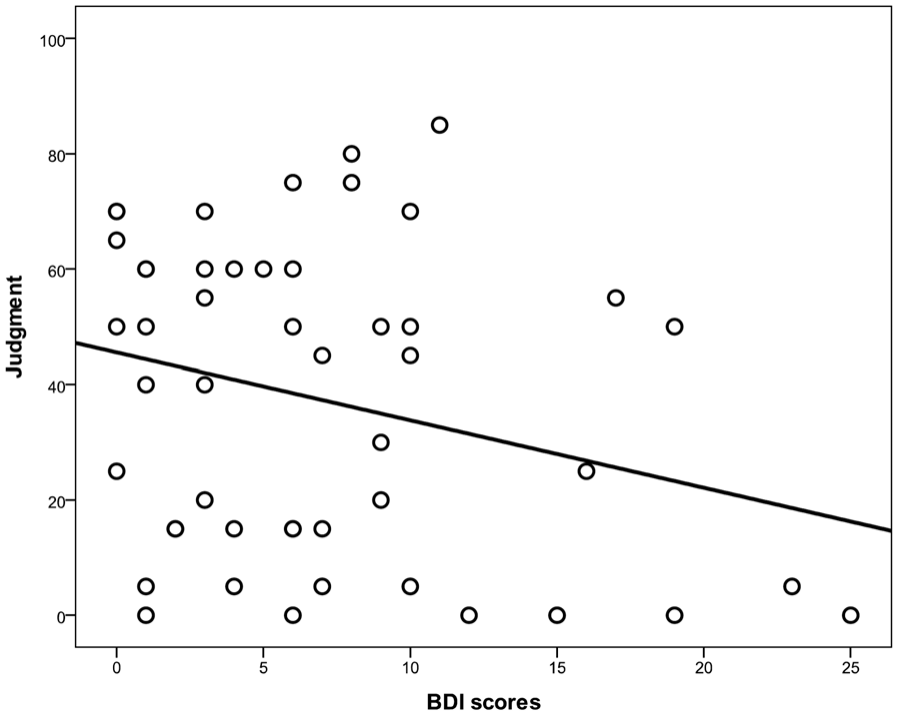
Scatterplot depicting the judgments as a function of BDI scores in Study 1. The scatterplot shows the judgments of control (vertical axis) as a function of the direct BDI scores (horizontal axis). The line fitting the data points displays a negative slope, indicating that the participants with higher BDI scores were more accurate in their judgments, thus replicating the depressive realism effect.

Table 1 contains the descriptive statistics (mean, standard error of the mean, range and median) for the three variables assessed: direct BDI score, P(R), and judgment of control. Instead of dichotomizing the continuous variables, as is usually done in this literature, we tested a potential mediational structure by means of the method described by Baron and Kenny [27]. In the proposed mediational structure, the BDI scores would affect the control judgments (i.e., produce the depressive realism effect) via the intermediate variable, as shown in Figure 1B. Following Baron and Kenny’s procedure, we first assessed the direct effect of the model, proving that BDI scores marginally predicted the judgments of control, *β* = −.27, *t*(48) = 1.95, *p* = .057 (Path c in Figure 1A). The negative coefficient implies that the higher the participant’s BDI score, the lower (and hence, more realistic) his or her control judgment was (see Figure 2). Therefore, we replicated the depressive realism effect described by Alloy & Abramson [1]. Next, we found that BDI scores also predicted the P(R), *β* = −.52, *t*(48) = 4.26, *p*<.001 (Path a in Figure 1B). The participants who were more depressed also responded less frequently during the task, in line with the findings of previous studies [9]. In the final step, we conducted a multiple regression analysis with BDI scores and P(R) as predictors and judgments as the predicted variable. This revealed that P(R) was a reliable predictor of the judgments even when controlling for the BDI scores, *β* = .40, *t*(47) = 2.57, *p*<.05 (Path b in Figure 1B), whereas the BDI scores failed to predict these judgments, *β* = −.06, *t*(47) = 0.40, *p* = .68 (the direct effect depicted as Path c’ in Figure 1B). The Sobel test [28] confirmed that the variance in the judgments that the BDI scores were able to predict (i.e., the total effect) was in fact accounted for by the indirect path via a mediator variable, P(R), *z* = 2.22, *p*<.05. Once the effect that the proximal cause, P(R), had on judgments was partialed out, there was little variance left in the judgments that could be predicted directly by the distal cause (namely, the BDI scores). This finding is consistent with our initial mediational hypothesis.

**Table 1:** Descriptive statistics.

		BDI scores	Judgments	P(R)
Study 1 (N = 49)	Mean	6.92	37.45	.75
	SEM	0.88	3.82	.03
	Range	0–25	0–85	.12–1
	Median	6	45	.88
Study 2: Analytic instructions (N = 95)	Mean	9.33	26.05	.48
	SEM	0.82	3.24	.03
	Range	0–36	0–100	.02–1
	Median	7	15	.48
Study 2: Naturalistic instructions (N = 103)	Mean	9.60	30.53	.65
	SEM	0.84	2.91	.02
	Range	0–40	0–100	.04–1
	Median	7	20	.70

## Study 2

**Figure 3:**
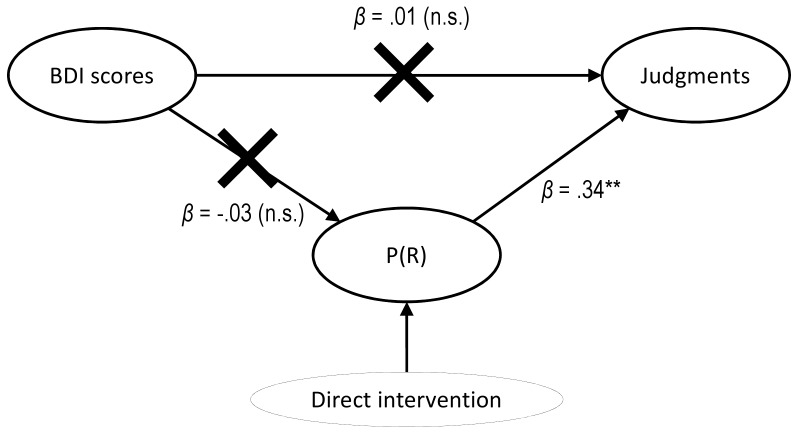
Diagram of the intervention in Study 2. The paths represent the links between the variables of interest, weighted by the provided standardized regression coefficients. One or two asterisks indicate a significant coefficient (*p*<.05 or *p*<.01, respectively), while n.s. means that the coefficient failed to reach the significance level (*p*>.05). In addition, crossed-out paths indicate that the two connected variables are not correlated to each other. First, the diagram shows that the direct intervention on the P(R) cancels out the otherwise natural effect of mood on activity level (i.e., the depressive passivity reported in Study 1 and in Blanco et al., 2009), as expected. Consequently, and given the mediational structure that we described in Study 1 (see Figure 1B), judgments are no longer predicted by BDI scores (i.e., the intervention renders the BDI scores and the judgments mutually independent, conditional on the mediator). This supports the mediational hypothesis in which the effect of mood on judgments is mainly due to the mediation of P(R).

Blanco et al. [9] and (more clearly) its exact replication in Study 1 of the current report both suggested that a causal chain model links depressive symptoms (as assessed by the BDI) to judgments of control via a mediator variable (the activity level), as shown in Figure 1B. To further investigate the mediation of the P(R) between the BDI scores and the judgments, we conducted Study 2. Here, we intervened by manipulating the P(R) via instructions, independent of depressive symptoms (as shown in Figure 3). A direct manipulation of the P(R) breaks the causal chain because it prevents mood from affecting P(R). Consequently, in Study 2, we should find that the P(R), and not the distal cause (BDI scores), is the variable that best predicts judgments of control.

This strategy aims to reveal causal links by means of interventions, and is grounded in the causal reasoning literature [18], [19], which typically embeds the variables into causal networks similar to those depicted in Figures 1 and 3. Under the mediational hypothesis, when an intervention is performed on a mediator variable the effect of the distal cause (called the “parent variable” in the causal networks literature) on subsequent elements (the “descendant variables”) of the chain is cancelled due to the Markov condition. Therefore, this manipulation serves as a test of the mediational hypothesis of depressive realism.

In addition, the choice of P(R) as the independent variable in Study 2 allows for disambiguation between our mediational hypothesis and an alternative causal model that may also be at the basis of the depressive realism effect. According to a common-cause model, both the P(R) and the control judgments would be directly affected by depressive symptoms. Crucially, the manipulation of the P(R) under such an alternative hypothesis would not eliminate the depressive realism effect because the intervention would be performed on a terminal node (i.e., a variable without descendants), and the link between BDI scores and judgments would remain statistically intact. That is, concerning the manipulation of P(R), the predictions of the two alternative models differ substantially.

### Method

#### Participants and apparatus

A sample of 209 anonymous Internet users volunteered to participate in the study through our laboratory’s webpage (www.labpsico.deusto.es). The experimental task was identical to that used in Study 1 and in previous publications [9], [20]. Previous studies conducted in our own laboratory and elsewhere showed that samples collected on the Internet are as reliable as those collected in the laboratory, specifically college students [20]. A frequently raised concern about Internet-based studies is the possibility of multiple submission of the same participant. We acknowledge that it is indeed impossible to assure that no participant in our sample took part twice in Study 2. However, according to most studies on this issue [29], [30], multiple submission is a rather uncommon event (of virtually negligible impact on the sample of 209 Internet users in Study 2) [29], [30]. Birnbaum [29] concluded that multiple submission “is not a real problem” in Internet-based research. Note also that, as mentioned above, we tested the reliability of this particular procedure (i.e., the flashes task) in previous studies in which we compared a sample of Internet users (collected in similar conditions to Study 2) with another sample comprising college students, and found the same results in both of them [20]. Moreover, convergent results concerning the depressive realism effect were also found in Blanco et al. [9], with a sample of Internet users, and in Study 1, which was conducted with college students, despite the fact that multiple submission was only possible through the Internet.

The participants were randomly assigned to one of two groups, which differed only in the instructions they received before the training phase. Eleven participants were excluded from the analyses because they did not respond in even a single trial during the experiment, which made the data they provided unreliable (there is no way to determine whether they were paying attention to the experiment). Therefore, the final sample was reduced to 198 volunteers, 103 in the Naturalistic Instructions group and 95 in the Analytic Instructions group.

#### Procedure

The procedure was identical to that used in Study 1 except for the instructions given before the training. The participants were presented with either of two instructional sets, the Naturalistic instructions or the Analytic instructions. The participants in the Naturalistic instructions group were encouraged to press the space bar frequently to make the flashes of light appear on the screen, whereas the participants in the Analytic instructions group were told to press the space bar in only 50% of the trials to determine whether they could control the flashes with their responses. The two instructional sets were based on the instructions from Matute [6], in which the same manipulation of the P(R) was conducted successfully.

### Results and Discussion

**Figure 4:**
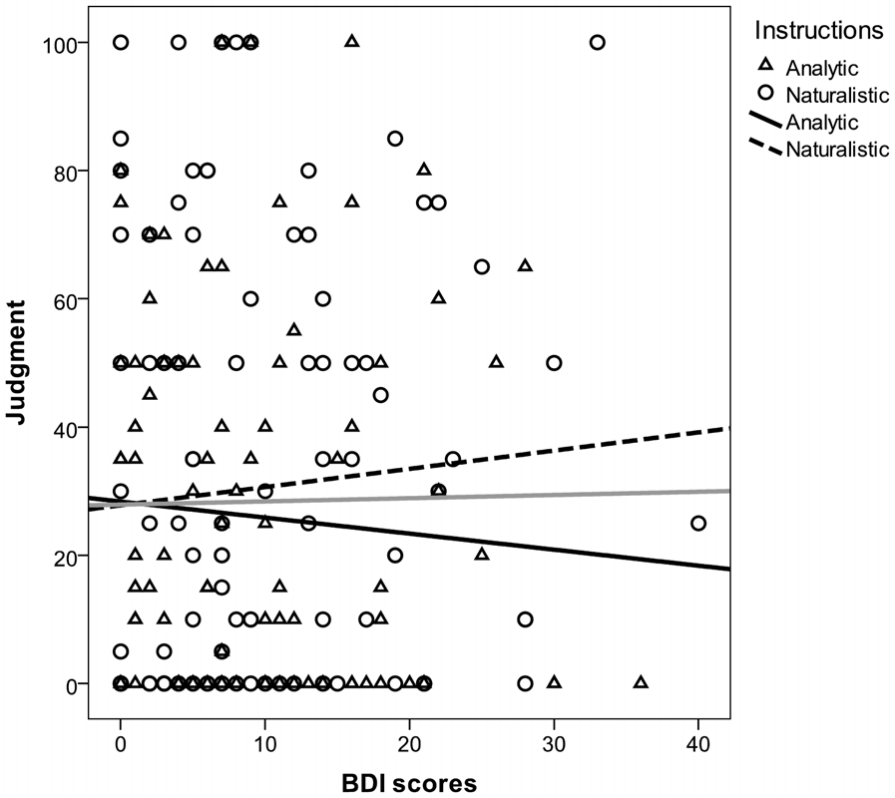
Scatterplot depicting judgments as a function of BDI scores in Study 2. Three regression lines are plotted: The shaded line shows the overall relation between BDI scores and judgments; the full black line and the dashed black line refer to the Analytic and Naturalistic groups, respectively. Note the flatness of the three slopes (in fact, no coefficient was significantly different from zero; see main text).

The descriptive statistics for BDI, P(R), and judgments are summarized in Table 1. To begin, we ensured that there were no significant differences in the BDI scores between the two groups (*F* <1), thus avoiding a potential source of sample bias. As planned, the instructional sets produced higher P(R) levels in the Naturalistic group than in the Analytic group, *F*(1, 196) = 21.15, *p*<.001. This manipulation was conducted independent of the participants’ depressive symptoms. As expected, the BDI scores were unable to affect the P(R) in Study 2, *β* = −.03, *t*(196) = 0.48, *p* = .63. The manipulation of the activity level successfully prevented the depressive passivity that we found in Study 1. In Figure 3, this is displayed as a crossed-out path between the BDI scores and the P(R). As we predicted from the mediational hypothesis, two linear regression analyses showed that BDI scores failed to predict the judgments, both when the two groups were collapsed, *β* = .01, *t*(196) = 0.19, *p* = .85, and when the instructions were controlled as a factor in the model, *β* = .01, *t*(195) = 0.17, *p* = .86 (observe the flat slopes in the scatterplot of Figure 4). Thus, intervening on the mediator variable P(R) resulted in the abolishment of the depressive realism effect.

In contrast, the P(R) was a good predictor of the judgments, *β* = .33, *t*(196) = 4.86, *p*<.001, even when we controlled for the instructions by entering them in the model, *β* = .34, *t*(195) = 4.75, *p*<.001. Moreover, once the P(R) effect was partialed out, the direct effect of the instructions on judgments was not significant, *β* = −.03, *t*(195) = 0.46, *p* = .64. This latter result indicates that the variance in the judgments was due to the P(R) rather than any potential direct influence of the instructions (e.g., different expectations directly induced by the different instruction versions).

To sum up, Study 2′s results converge with the conclusions from Study 1 by highlighting that P(R) predicts the variance in the judgments to a greater extent than the BDI scores do. This is consistent with the idea that P(R) is the proximal factor that mediates between depressed symptoms and judgments of control. Consistent with this mediational hypothesis, when we intervened directly on the P(R), the mood measure was unable to account for any variance in the judgments. Furthermore, the latter finding is not predicted by the alternative hypothesis in which both P(R) and judgments are directly dependent on the depressive symptoms.

## General Discussion

The intriguing finding by Alloy and Abramson [1] opened a fertile research field that soon spread from basic learning literature to social and applied psychology (see reviews [13], [14], [31], [32] and a recent meta-analysis [33]). According to the numerous works published on the topic, many different variables may modulate the depressive realism effect, which explains why cannot always be replicated. Some of the previously studied variables include the valence of the outcomes [1], [34], the presence of an observer [35], the outcome frequency [1], and the length of the intertrial interval [10], [11]. Recently, Blanco et al. [9] suggested that yet another factor contributes to the so-called depressive realism effect. As they pointed out, depressed participants’ lack of responding during the training phase prevents the illusion of control by reducing the frequency of accidental concurrences between the participant’s responses and the uncontrollable outcome. In contrast, the nondepressed participants (who are usually more active and respond frequently during the training phase) are prone to develop an illusion of control via the formation of a spurious association between the two events (see Blanco et al. [15] and Matute [6] for demonstrations of the effect of the response frequency on the illusions of control). Not only is the P(R) effect a robust empirical finding, it is also consistent with the predictions of leading associative learning theories, e.g., the Rescorla-Wagner model [36]; see Matute, Vadillo, Blanco, and Musca [37] for computer simulations illustrating this point).

Blanco et al. [9] showed that, in an uncontrollable task, depressed participants were both more passive and more accurate than nondepressed participants, while the response frequency correlated positively with the illusion of control. In Study 1, we conducted an exact replication of Blanco et al. [9] featuring a mediational analysis aimed at revealing the causal structure connecting the three variables of interest. This analysis confirmed that higher BDI scores correlated with more accurate judgments of control (i.e., depressive realism) and that probability of responding, P(R), mediated the effect between depressive symptoms and judgments. The resulting causal model can be depicted as a chain, with BDI scores as the distal cause and the P(R) as a more proximal cause of the variance in the judgments of control (Figure 1B). Crucially, depressive symptoms affected the judgments only via the mediator variable, P(R). In addition, while the study conducted by Blanco et al. [9] used a sample of anonymous Internet users, Study 1 in the current report was conducted with college students, and both yielded similar results (the former population could be assumed to be relatively heterogeneous in age, sex, etc.). In summary, the results from Study 1 suggest that the findings of Blanco et al. [9] are robust and partially generalisable across populations, while complementing them with additional statistical evidence of the mediator role of P(R) in the depressive realism effect.

Study 2 provides converging evidence for the latter finding. The same causal structure was tested, but through a different strategy (experimental intervention). Study 2 used exactly the same tasks and parameters that were used in Blanco et al.’s work [9] and Study 1, except for the instructions that the participants received before the training phase. These instructions were intended to manipulate the probability of responding, in a manner similar to that employed in previous studies [6]. As expected, the group that received the Analytic instructions (which included the requirement to respond on 50% of the trials) responded less frequently than the group that was presented with the Naturalistic instructions, which included encouragement to respond frequently.

Aligning with Blanco et al. [9], the only predictor of judgments was the probability of responding. Thus, when we prevented depressive symptoms from influencing the level of responding (because participants were explicitly instructed to respond with either high or low frequency), it was the response frequency and not the depressive symptoms that predicted the judgments: A higher probability of responding led to a stronger illusion of control, whereas a lower probability of responding led to better accuracy at detecting the uncontrollability of the outcome. We highlight that, apart from the instructional manipulation, the task was identical to the one in which the depressive realism effect was shown in Blanco et al. [9] and in Study 1. In addition, the sample size of Study 2 (*N* = 198) was much larger than that of Study 1 (*N* = 49) and Blanco et al. (*N* = 66), hence minimizing the possibility that the absence of an effect of depressive symptoms was due to a lack of statistical power. Then, we conclude that depressive realism vanished when depressive symptoms were not allowed to influence the response frequency because the latter was directly manipulated.

It is important to note that our account that the depressive realism effect is mediated by the probability of responding is not incompatible with other proposed mechanisms based on cognitive or behavioral factors. Thus, it is not intended to rule out the complex and multicausal view on the phenomenon that some experts support [13]. The clear and distinctive prediction that can be derived from our proposal is that any manipulation aimed at reducing the frequency of responses should also lead to the more accurate detection of uncontrollability, be it depressive symptoms (as in Study 1 and Blanco et al. [9]), instructions (as in Study 2), fatigue, motivational factors, an analytical rather than naturalistic approach to the problem, or other means.

Moreover, our mediational hypothesis allows a theoretical account of depressive realism in terms of contingency learning. It is important to mention that depressive realism has been recently studied in this domain [10], [11], [14]. Most theoretical models of learning, such as the Rescorla-Wagner model [36], do not include any explicit statement about the effects of motivational or mood-related factors on the learning of associations between stimuli. If these factors are reconceptualized, however, as distal causes mediated by the P(R), as we argue here, then some contingency learning models may indeed account for depressive realism and related phenomena because the effect of the mediator variable, P(R), can be easily addressed by these theories. For instance, some contingency learning models would claim that the illusion of control displayed by nondepressed/active participants in our studies could be explained as the result of granting uneven importance to different types of trials during the training phase (i.e., cell weighting). That is, when evaluating contingency, the participants give different weights to each type of events they are exposed to, and usually consider concurrences of the desired outcomes with their responses to be the most important event [38]–[40]. In more general domains (e.g., social psychology), people are also known to base their beliefs and decisions on confirmatory evidence, neglecting disconfirmatory information [41], [42] in a way similar to the one we are describing here. This idea is also implicit in the Rescorla-Wagner model [36], given the widely accepted assumption that the learning rate parameter possesses a greater value in response-present trials than in response-absent trials. As a result of this asymmetry, the associative strength of the response increases more rapidly because of the accidental concurrences than it decreases because of the response-no outcome trials. Furthermore, note that for those participants responding with high probability, the accidental response-outcome concurrences are actually the most frequent type of trial [37].

To sum up, the predictions of these two variants of the contingency learning theory (cell weighting-based and associative) converge when they are applied to our two current studies: They both predict that increasing the response frequency will strengthen the illusory perception of a response-outcome correlation (assuming that the probability of the desired outcome is high in all cases). Thus, a mediational hypothesis that transfers motivational or depression-related differences to a more proximal cause, P(R), which is amenable to modeling in contingency-learning terms (unlike the former variables), becomes useful for making specific or quantitative predictions and modulating the depressive realism effect.

Finally, we would like to discuss the implications of our current findings for the clinical field. It has been argued that an accurate and realistic perception of reality is not always a healthy feature [8], [29]. In fact, the illusion of control protects against stressors, both natural and artificially induced in the laboratory [8]. Therefore, some theories claim that certain optimistic biases and illusions are attributes of healthy, well-adapted behavior [29], [43], [44]. According to this view, the illusion of control may enhance self-esteem, leading the individual to optimistically believe that he or she is able to control certain aspects of his or her life instead of attributing them to chance or to uncontrollable factors. It has been suggested that depressed people are not as motivated to protect their self-esteem as healthy people are, leading to the realistic but uncomfortable perception of uncontrollability [43], [44].

Based on the mediational hypothesis presented here, we can suggest that clinically imposed contingencies aimed at increasing the depressed patient’s activity and probability of responding (like those commonly used in cognitive-behavioral therapy) may help develop these healthy, optimistic illusions. Therapists in the behavioral tradition are already promoting an increase in patient activity as a clinical strategy against depression [17], [45]. These therapies are mostly based on theories that conceptualize depression as a result of a previous history of nonreinforcement, and being active is normally the best possible way to obtain reinforcers. We acknowledge the obvious limitations inherent in working with nonclinical samples, such as college students or Internet users; still, we note that our results tend to coincide with the previously mentioned approach to depression treatment, and they add value to it: Increasing the probability of responding is good not only because it allows people to obtain controllable reinforcers but also because it makes their behavior coincide even with those desired outcomes that occur by mere chance and are beyond the individual’s control, thus leading to the development of healthy optimistic illusions.
